# DScan – a high-performance digital scanning system for entomological collections

**DOI:** 10.3897/zookeys.209.3115

**Published:** 2012-07-20

**Authors:** Stefan Schmidt, Michael Balke, Stefan Lafogler

**Affiliations:** 1 Zoologische Staatssammlung, Münchhausenstr. 21, 81247 Germany; 2 Technisches Büro München, Thierschstr. 20, 80538 München, Germany

**Keywords:** Entomology, insect collection, insect drawer, CNC technology

## Abstract

Here we describe a high-performance imaging system for creating high-resolution images of whole insect drawers. All components of the system are industrial standard and can be adapted to meet the specific needs of entomological collections. A controlling unit allows the setting of imaging area (drawer size), step distance between individual images, number of images, image resolution, and shooting sequence order through a set of parameters. The system is highly configurable and can be used with a wide range of different optical hardware and image processing software.

## Introduction

Natural history collections are nature’s treasure houses. About 80 million objects are deposited in German natural history collections alone, including about 65 million insects (Brake and Lampe 2004). The Zoologische Staatssammlung in Munich, Germany (ZSM) holds about 25 million zoological objects. About 90% of the collection are insects, including 10 million Lepidoptera, 3-4 million Coleoptera, and about three million Hymenoptera, stored in about 100,000 standard sized drawers (51 × 42 cm).

The material deposited in natural history collections like the ZSM is principally held and intended to support research purposes. Natural history collections are indispensable scientific resources that play a central role in biodiversity research (Wheeler et al. 2012). However, the level of documentation of entomological collections is very low, and even basic data about specimens or metadata about collections are often completely missing (Brake and Lampe 2004). Moreover, digitisation of natural history specimens is labour-intensive and usually proceeds at a very slow pace. This is partly due to a regrettable lack of personnel, a situation that is not going to change in the foreseeable future. Technical solutions have the potential to aid our digitisation efforts by reducing the need for extensive human resources. However, these solutions need to be developed. Our aim is to use innovative approaches to develop new methods for the rapid digitisation of entomological collection drawers, and the subsequent extraction of relevant metadata from drawer images.

DScan is a prototype scanning machine and the foundation of a digitisation system that allows fast and efficient digitisation of entomological drawers. Our primary aim is the optimisation of this system for on-demand-digitisation requirements. Because the contents of and arrangement of specimens within drawers will change if they are part of an active research collection, re-scanning of drawers needs to be as fast and as easy as possible.

The resulting images allow inspection of insect specimens at high resolution without the need to access the collection itself physically. The level of detail can be adjusted as required, for instance in relation to the size of the insect specimens, and, is in most cases sufficient for specialists to recognize the taxon at genus or even species level.

## Mechanics of the drawer scanning system

**Figure 1.**
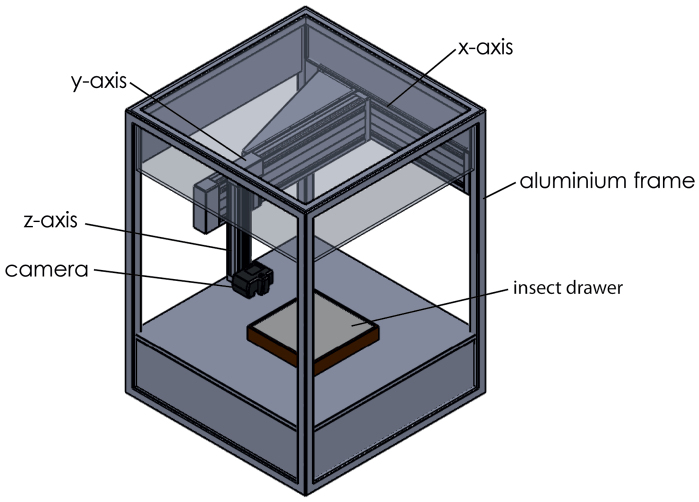
Schematic drawing of the DScan system. Flashes (not shown) are placed inside the scanner.

DScan is made of a sturdy, industrial standard aluminium frame (LWH = 1080 × 1080 × 1500 mm) with linear units as used by Computer Numerical Control (CNC) positioning machines (Fig. 1, and YouTube video under youtu.be/zyT7l-CZego). Servo drives and precision ball screw spindles allow a minimum step distance of 0.02 mm at a maximum speed of 100 mm/s. Effective travel ranges are 600 × 600 mm horizontally (x- and y-axis) and 200 mm vertically (z-axis). The system is operated by a PC-controlled console (netbook) with ProNC software (DNC Software Ltd, www.pronc.com). The left and right sides and back of the scanner are covered by white panels. The front is closed by a curtain with a reflective inner surface that is closed during scan operations.

### Optics

Choice and selection of the optical components of the drawer scanning system are largely unconstrained by the mechanics of the positioning components of the DScan mechanism. A wide range of different camera systems can be adapted to work with the DScan, provided that the camera has remote control capability because the shutter release needs to be triggered by the control unit. Currently the best option includes a digital single-lens reflex (DSLR) camera, although the recent introduction of mirrorless system cameras with interchangeable lenses and comparatively large sensors will increase the range of suitable optical equipment. In addition, mirrorless cameras employing electronic shutter mechanisms avoid the wear that can be significant when using a DSLR for creating large numbers of images.

Our current system comprises a Nikon D300 DSLR camera equipped with a 12 megapixel APSC sensor, attached to a Voigtländer 90 mm Apo-Lanthar macro lens. As light sources we use two studio flash lights that are placed inside the scanner compartment. The white inner surfaces of the top and side panels, and the white front curtain are highly reflective and allow for maximum lighting efficiency. The flashes are directed toward the side and top panels to achieve an even and non-reflective illumination of specimens. The indirect lighting reduces the risk of blown-out highlights caused by reflections from insects with smooth surfaces and exceedingly high contrast. Typically, these effects are caused by direct, punctual lighting when photographing insects with strongly sculptured and shining, in particular metallic, surfaces.

### Scanning process

The system takes images of a single drawer in a sequential order as determined by the controlling unit. The shooting order is customizable and can be configured by modifying parameters of the control program. The best results are achieved when each photograph overlaps its neighbours by about 30-40%, which enables the stitching software to generate smooth transitions between images. The number of images per drawer can be adjusted by changing the distance between drawer and camera (z-axis). A lower z-distance results in a larger number of images and a larger size and higher resolution of the final megapixel image. A full scan of a standard sized drawer (51 × 42 cm) at a distance of 60 cm takes about 2.5 minutes and produces a set of 56 images (see the DScan in action on YouTube, youtu.be/zyT7l-CZego). At a distance of 52 cm, which is the minimum distance of the macro lens we are using without close-up lens, a scan comprises 99 images and the scanning process takes about 4.2 minutes.

### Image processing

**Figure 2.**
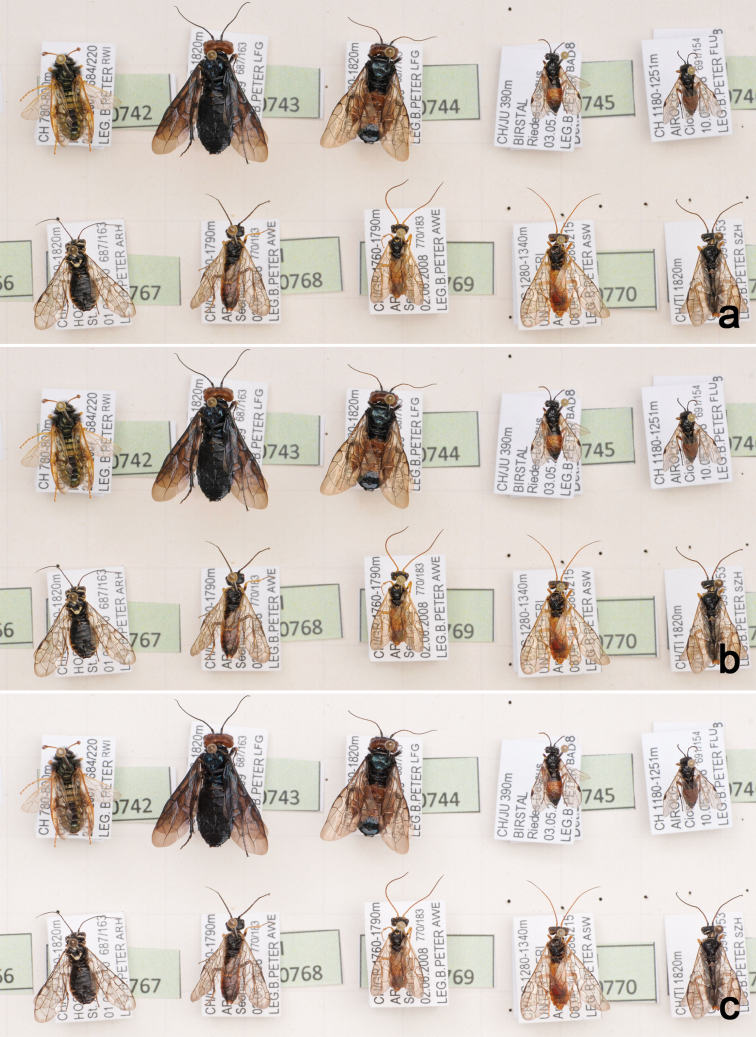
Partial drawer images taken at the same position using three different file formats: captured as JPEG (**a**), captured as TIFF (**b**), and TIFF converted from RAW (**c**). A high resolution version of the image is available under media.zsm-entomology.de/suppl/zookeys_mass_digitisation_volume/Fig_2.png

**Figure 3.**
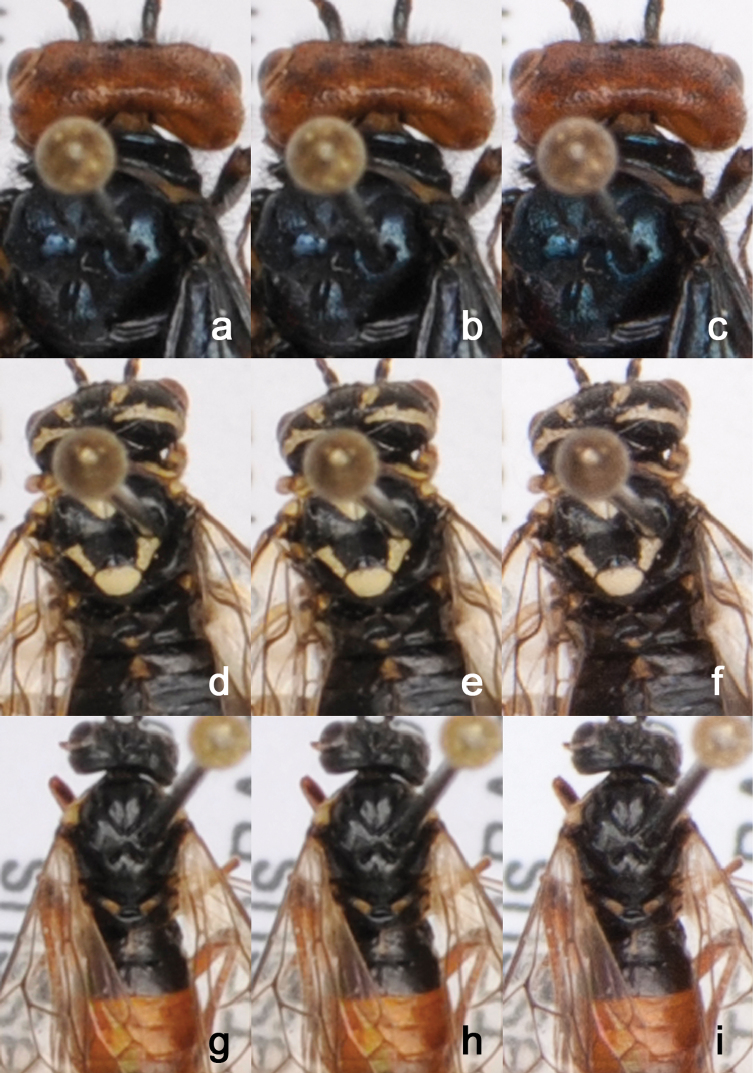
Enlargements of drawer images from Fig. 1 to show quality differences between image file formats. Each of the three specimens was captured in JPEG (**a, d, g**), TIFF (**b, e, h**), and RAW (**c, f, i**). A high resolution version of the image is available under media.zsm-entomology.de/suppl/zookeys_mass_digitisation_volume/Fig_3.png

To obtain the final high-resolution image, the captured images from each drawer need to be assembled or “stitched” using dedicated stitching or panorama software. For this purpose, we use AutoPano Giga (Kolor, www.kolor.com). Images are captured in RAW format, developed using Capture NX2 (www.capturenx.com) and saved as 8- or 16-bit TIFF images. Alternatively, images can be captured in JPEG (Figs 2a; 3a, d, g) or TIFF format (Figs 2b; 3b, e, h) and directly assembled without the need of image development. Using JPG format reduces the post processing effort but produces images of slightly inferior quality compared to RAW (cf. Fig. 3a, d, g vs 3c, f, i, for high resolution versions of Figures 2 and 3 see media.zsm-entomology.de/suppl/zookeys_mass_digitisation_volume/Fig_2.png and media.zsm-entomology.de/suppl/zookeys_mass_digitisation_volume/Fig_3.png).

With 56 images per drawer, the final stitched image has a size of ca. 300 megapixels, whereas images that are assembled from 99 photographs result in pictures of about 500 megapixels. The resulting images are far too large for display in a web browser and need to be made “zoomable” by tiling and creating a low resolution version of the original image. This can be achieved by dedicated software such as Zoomify (Zoomify, Inc., www.zoomify.com) or Krpano (krpano GmbH, krpano.com). During this process, tiles are created at different resolutions, allowing zoom-and-pan viewing of the drawer image so that if parts of a drawer image are enlarged, the corresponding tiles are loaded, thus avoiding the need to load the full high-resolution image before it can be viewed. Sample images are available online at zsm-entomology.de and show insect drawers containing Coleoptera (zsm-entomology.de/wiki/Drawer_Digitization_Project_-_Coleoptera), Hymenoptera (zsm-entomology.de/wiki/Drawer_Digitization_Project_-_Hymenoptera), and Lepidoptera (zsm-entomology.de/wiki/Drawer_Digitization_Project_-_Lepidoptera).

### Performance

With an optimised workflow in place, from capturing individual images of a single drawer to the final megapixel image, processing of about 100 insect drawers per day seems technically possible. This assumes a scan rate of 20 drawers per hour plus 10 hours for processing the images in batch mode, which can be done overnight. Image processing (developing, stitching, and image adjustment) depends largely on the computer hardware used. Using a workstation equipped with two Intel Xeon processors with 12 MB cache and a speed of 2.26 Ghz each, 24 MB of RAM, and a high-end graphic card, the stitching process of 56 individual TIFF images using the software AutoPano Giga takes about 4.5 minutes. The subsequent generation of multi-resolution images in jpeg format using Krpano takes an additional 1.5 minutes, resulting in a total of about 6 minutes for the computational part of the scanning process, starting from a set of individual images to high-resolution, zoomable images that are ready for dissemination on the internet.

### Costs

The costs for the CNC system itself without optics, computer hardware, and software amount to about USD $25,000. The camera system, i.e. a digital SLR with macro lens and studio flash lights, comes to about $2,000–$3,500, adding up to about $30,000 in total for the system including software (AutoPano Giga, krpano) but without computer hardware for image processing and storage. However, as mentioned before, available hardware can be used and a range of suitable cameras can be fitted to the system, requiring only minor modifications to the controlling unit and cable connectors. Standard computer hardware can be used for the stitching of images although the processing will take longer than with a dedicated workstation.

### Further developments and prospects

**Figure 4.**
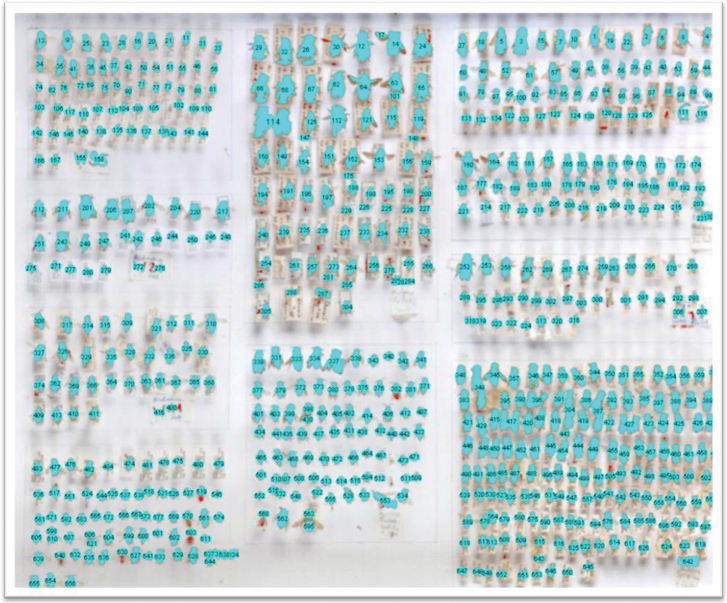
Automatic numbering of specimens using ImageJ. For details see text.

The DScan system aims to achieve rapid digitisation of entomological collections. The high-resolution images of insect drawers themselves contain a wealth of information. However, additional processing is required to extract that information from the images and make computable metadata about the drawer content available and searchable. Currently, we generate basic metadata associated with each drawer manually, including taxon information and geographic coverage. Several ways to extract metadata from drawer images are currently being explored and evaluated:

• Counting the number of specimens in a drawer and at the same time assigning numbers to each specimen using image analysis software like ImageJ (http://rsbweb.nih.gov/ij/) (Fig. 4). The number and position of each specimen can be exported and used, for instance, for image analysis purposes. For example, the position (x-, y-coordinates) of specimens can be used to automatically crop an image around the position of a specimen. This would allow to create individual images of specimens in a drawer.

• Adding a clickable “hot spot” to individual specimens in a drawer that shows, when selected, specimen- or species-related information, for example, type data. The trigger can also be used to open a text box, open an external web site, or submit a database query.

• There could be an option for users to interactively mark certain specimens, for example taxonomists who would like to borrow certain specimens for closer examination. Additionally, users may be given the opportunity to add information to specimens, e.g., identifications.

• Metadata could be extracted using Optical Character Recognition software.

• Specimens with a Quick Response (QR) code label that is visible from above can be tracked in a collection.

• Cutting out individual specimens from the drawer image and associating metadata with them will bridge the gap between digitisation at drawer and at specimen level.

• Extended depth-of-field photography to avoid parts of the image (e.g. bottom labels) being out of focus. This is particularly important when depth-of-field becomes very short at close object distance.

The above list includes only some ideas that seem worthy of exploration in the future. More applications and analysis methods will surely emerge once the system is used routinely, provided that funding opportunities permit further development.
